# Pathological similarities between low birth weight-related nephropathy and nephropathy associated with mitochondrial cytopathy

**DOI:** 10.1186/s13000-014-0181-0

**Published:** 2014-09-30

**Authors:** Toshiyuki Imasawa, Masashi Tanaka, Naoki Maruyama, Takehiko Kawaguchi, Yutaka Yamaguchi, Rodrigue Rossignol, Hiroshi Kitamura, Motonobu Nishimura

**Affiliations:** Kidney Center, National Hospital Organization Chiba-East Hospital, 673 Nitona-cho, Chuoh-ku, Chiba-city, Chiba 260-8712 Japan; Clinical Research Center, National Hospital Organization Chiba-East Hospital, Chiba-city, Chiba Japan; Department of Genomics for Longevity and Health, Tokyo Metropolitan Institute of Gerontology, Itabashi, Tokyo Japan; Aging Regulation Section, Tokyo Metropolitan Geriatric Hospital and Institute of Gerontology, Itabashi, Tokyo Japan; Yamaguchi Pathology Laboratory, Matsudo, Chiba Japan; EA4576 MRGM, University of Bordeaux, Bordeaux, Gironde France

**Keywords:** Focal segmental glomerulosclerosis, Low birth weight, Mitochondria, Granular swollen epithelial cell

## Abstract

**Background:**

Individuals born with a low birth weight (LBW) have a higher risk of developing kidney dysfunction during their lifetime and sometimes exhibit focal segmental glomerulosclerosis (FSGS) lesions in their glomeruli. We herein try to obtain other pathological characteristics of LBW-related nephropathy.

**Methods:**

We retrospectively evaluated the renal pathology of four patients demonstrating FSGS with a history of LBW. Two mitochondrial cytopathy patients were also analyzed. DNA mutations were surveyed using a PCR-Luminex assay.

**Results:**

In all four FSGS patients with a history of LBW, focal segmental glomerulosclerosis were detected. Interestingly, granular swollen epithelial cells (GSECs), which have previously been reported exclusively in patients with mitochondrial cytopathy, were also observed in the distal tubules and/or collecting ducts of all four patients with a history of low birth weight in this study. Electron microscopy revealed that these granular swollen epithelial cells included an increased number of enlarged mitochondria. Furthermore, cytochrome c oxidase subunit IV staining of patients with a history of low birth weight and patients with mitochondrial DNA mutations showed unbalanced expression patterns in glomeruli and a part of tubular cells. However, no mitochondrial gene mutations were detected in any of our four patients with low birth weight-related nephropathy.

**Conclusions:**

This is the first report to show the pathological similarities not only in glomeruli but also tubuli between nephropathy with a LBW history and nephropathy with mitochondrial cytopathy.

**Virtual Slides:**

The virtual slide(s) for this article can be found here: http://www.diagnosticpathology.diagnomx.eu/vs/13000_2014_181

## Background

Low-birth-weight (LBW) is also associated with an increased risk of end-stage renal disease (ESRD) [1]. Studies of both humans and animals have shown LBW to be significantly associated with a decreased number of nephrons [2-4]. Brenner proposed the glomerular hyperfiltration theory in which adaptive mechanisms activated in response to nephron loss increases the capillary pressure and the incidence of glomerular hypertrophy [5,6]. This intraglomerular hypertension results in accelerated damage to nephrons with further nephron loss. This vicious cycle promotes the further progression of chronic kidney disease (CKD).

In particular, it is well known that representative glomerular changes in LBW individuals include focal segmental glomerulosclerosis (FSGS) [7]. Although intraglomerular hypertension is associated with the pathogenesis of FSGS, the clear mechanisms by which FSGS lesions are formed have not been clarified. Because detail pathological analysis sometimes gives a clue to assess the pathogenesis, we herein evaluated LBW-related nephropathy (LBWN) in order to obtain more detail pathological characteristics.

## Methods

### Patients

From January 2006 to December 2011, we performed 472 kidney biopsies in our division. In these cases, there were four cases of FSGS among patients born with a birth weight under 2,500 g (according to the WHO definition of LBW). These four patients (LBW 1–4) were retrospectively evaluated pathologically and genetically. In addition, two patients (Mt 1 and 2) who were found to have mitochondrial DNA (mtDNA) mutations and underwent kidney biopsies to investigate the cause of their proteinuria were also evaluated. As normal controls for staining (N = 3), kidneys dissected because of kidney cancer were used.

### Histological analysis

Briefly, kidney specimens fixed in 10% neutral buffer formalin followed by embedding in paraffin were used for a light microscopy analysis with routine staining, as follows: hematoxylin and eosin (HE), periodic acid-Schiff (PAS), Masson’s trichrome stain and periodic acid-methenamine-silver (PAM)-HE stain. The kidney specimens used for the electron microscopy analysis were fixed in 2% glutaraldehyde (pH 7.3-7.4) followed by 2% osmium tetroxide (pH 7.3-7.4). For cytochrome c oxidase subunit IV (COX IV) staining, paraffin-embedded specimens were used. Following deparaffinization and antigen activation using 10 mM of boiled citrate buffer (pH 6.0), rabbit anti-COX-IV antibodies (clone 3E11) (Cell Signaling Technology, Inc., Danvers, MA) were used as the primary antibody. SignalStain® Boost IHC Detection Reagent (HRP, Rabbit) (Cell Signaling Technology, Inc.) was used to detect the rabbit primary antibody, according to the manufacturer’s instructions.

### Assay for detecting mitochondrial gene mutations

Blood samples (1 ml/each analysis) were collected in EDTA-2Na tubes. Following centrifugation, the pellets were used for the analysis. For the analysis of urine sediments, over 100 ml of urine was collected from each patient, and the pellets obtained after centrifugation were used for the analysis. Five slices (4 μm) of frozen kidney sections were used for the genetic analysis. A PCR-Luminex assay, which can be used to survey 61 different pathogenic mtDNA mutations, was performed according to our previously reported method [8].

### Ethical considerations

This study was conducted in accordance with the “Ethical Guidelines for Clinical Studies” (Revised on December 28, 2004, Ministry of Health, Labour and Welfare of Japan). All medical professionals involved in this study were required to comply with these ethical standards. The local Ethics Committee of Chiba-East Hospital approved the study protocol (No. 22), and all subjects provided their informed consent to participate in this study.

## Results

### All four LBW-related nephropathy patients exhibited perihilar variants of FSGS in their glomeruli

**Figure 1:**
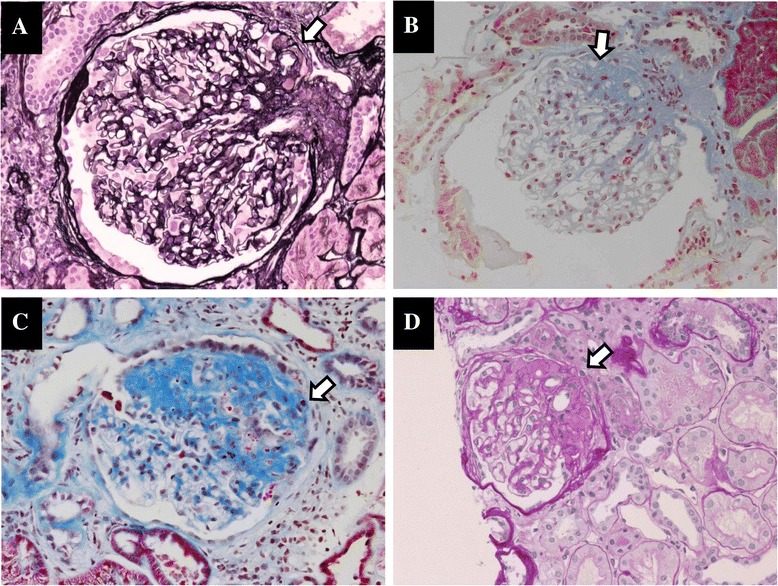
**Representative photos of glomeruli in the FSGS patients born with LBW. (A)**: Exudative and sclerotic lesions are indicated in the perihilar area by arrows (LBW 1, PAM-HE stain). **(B)**: Segmental sclerosis in the perihilar area is indicated by the arrow (LBW 2, Masson’s Trichrome stain). **(C)**: Segmental sclerosis is indicated by the arrow (LBW 3, Masson’s Trichrome stain). **(D)**: Segmental sclerosis in the perihilar area is indicated by the arrow (LBW 4, PAS stain).

**Figure 2:**
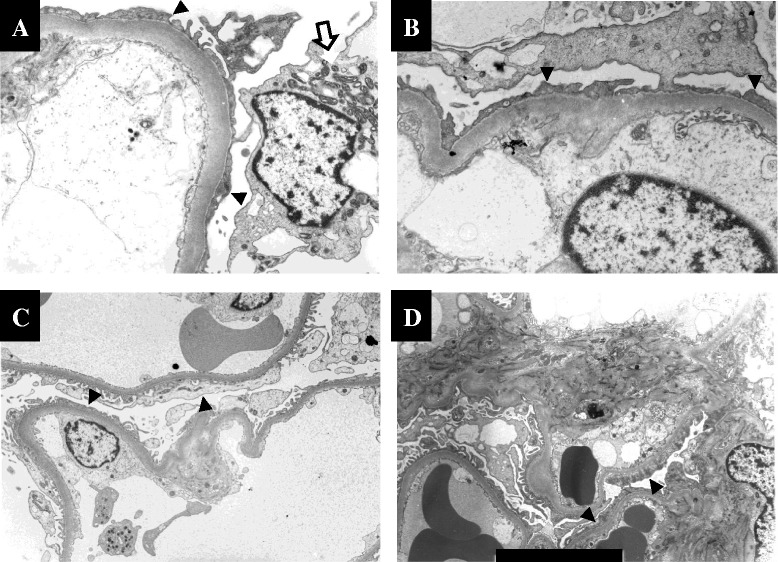
**Electron microscopy of the glomeruli. (A)**: In Mt 1, a glomerular podocyte (indicated by the arrow) exhibits an increased number of mitochondria. The podocytes show patchy foot process effacement (arrowheads). **(B-D)**: In LBW 1 **(B)**, LBW 2 **(C)**, and LBW 3 **(D)**, the number and morphology of mitochondria in the glomerular podocytes are normal. However, the podocytes show patchy foot process effacement (arrowheads).

The background characteristics of the patients are summarized in Table 1. All four LBWN patients underwent kidney biopsies in order to determine the etiology of their persistent proteinuria. All four LBWN patients were male, with a mean birth weight of 1,692 g. The gestational ages and the reasons for the low birth weight could not be fully assessed in these cases (The gestational age of LBW1 was 28 weeks and that of LBW2 was 38 weeks). No subjects had symptoms of myopathy or encephalopathy, a history of stroke-like episodes or difficulty hearing. Although all patients had no subjective symptoms, they were found to have proteinuria on periodic medical examinations. Only one (LBW 3) of the LBWN patients suffered from diabetes mellitus (with a five-year history). The kidney size tended to be smaller than normal, except in LBW 3 (Table 1). The light microscopy analysis revealed that the glomeruli of all LBWN patients exhibited FSGS (Figure 1). Patient Mt 1 was initially found to have proteinuria at 39 weeks of gestation and was subsequently referred to our hospital because the proteinuria persisted for one year after delivery. Although she had no symptoms of MELAS (mitochondrial myopathy, encephalopathy, lactic acidosis, stroke-like episodes), we found increased morphologically abnormal mitochondria in her podocytes (Figure 2A). Therefore, we evaluated her mtDNA at our hospital and detected an mtDNA mutation (3243 A > G). Patient Mt 2 was found to have proteinuria on a periodic medical checkup at 19 years of age. She also had no symptoms of MELAS. She refused an assessment of her serum lactate level. However, she had a brother with MELAS (3243 A > G), and a kidney biopsy revealed that her podocytes included increased mitochondria (data not shown). Her mtDNA mutation (3243 A > G) was diagnosed at another hospital. Although the characteristic glomerular change in patients with mitochondrial cytopathy is FSGS, as previously described in several reports [9-11], neither of our two patients with mtDNA mutations had apparent FSGS lesions in the evaluated glomeruli (data not shown). An electron microscopy analysis of the two patients with mtDNA mutations revealed that the glomerular epithelial cells contained an increased number of enlarged mitochondria and mild foot process effacement. On the other hand, we found no morphological abnormalities or increments in the number of mitochondria in any of the observed sections obtained from the LBWN patients (Figure 2B-D). However, the podocytes of the four LBWN patients showed mild foot process effacement (Figure 2B-D), similar to that observed in the patients with mtDNA mutations (Figure 2A).

**Table 1:** **Summary of patients**

**Case**	**Age**	**Sex**	**BMI** ^**1**^	**Birth weight (g)**	**BP** ^**2**^ **(mmHg)**	**eGFR** ^**3**^ **(ml/min/1.73m** ^**2**^ **)**	**Urinary protein** ^**4**^ **(g/gCr)**	**Serum lactate** ^**5**^ **(mg/dl)**	**Long axis of kidney** ^**6**^ **(mm)**	**Number of total glomeruli**	**Global sclerosis** ^**7**^ **(%)**	**Segmental sclerosis** ^**8**^ **(%)**	**Glomerular hypertrophy** ^**9**^	**Foot process effacement**	**mtDNA mutation** ^**10**^
LBW1	21	M	22.0	978	120/65	98	0.34	15.2	880	24	8.3	16.7	+	+	none
LBW2	20	M	21.0	1900	126/82	46	0.56	n.d.	848	4	0	25	+	+	none
LBW3	36	M	31.2	1400	125/75	74	1.35	n.d.	1208	14	42.9	7.1	+	+	none
LBW4	41	M	24.5	2465	121/70	63	0.83	15.8	988	20	15	10	+	+	none
Mt1	34	F	17.6	2720	115/63	48	0.29	9.1	936	11	45.5	0	-	+	3243 A>G
Mt2	28	F	17.5	3630	96/63	118	1.39	n.d.	953	16	0	0	-	+	3243 A>G

^1^BMI, body max index at the time of kidney biopsy.

^2^BP, blood pressure: BP at the time of kidney biopsy is expressed as systolic/diastolic.

^3^eGFR, estimated glomerular filtration: eGFR is calculated by Japanese GFR equation [12] from serum creatinine value, age, and sex at the kidney biopsy.

^4^The data of urinary protein is based on a urinalysis on the day of the kidney biopsy.

^5^Serum lactate is measured by an enzymatic assay (normal range: 3-17mg/dl). We did not measure the lactate values in three of six patients (expressed as “n.d.”).

^6^The long axis of the left kidney was measured by an echogram just before the kidney biopsy.

^7^The rate of the number of globally sclerosed glomeruli/total glomeruli in the observed section is expressed.

^8^ The rate of the number of segmentally sclerosed glomeruli/remnant glomeruli (total glomerular number minus GS glomeruli) is expressed.

^9^The existence of glomerular hypertrophy is defined when the diameter of capillary area is over 250μm [13].

^10^mtDNA mutation was surveyed in blood, urine, and kidney specimens by the PCR-Luminex method [11] except Mit2 patient, who was already diagnosed by a gene analysis in the other hospital.

### All four patients with LBW-related nephropathy and the two patients with mtDNA mutations had granular swollen epithelial cells in their collecting ducts or distal tubules

**Figure 3:**
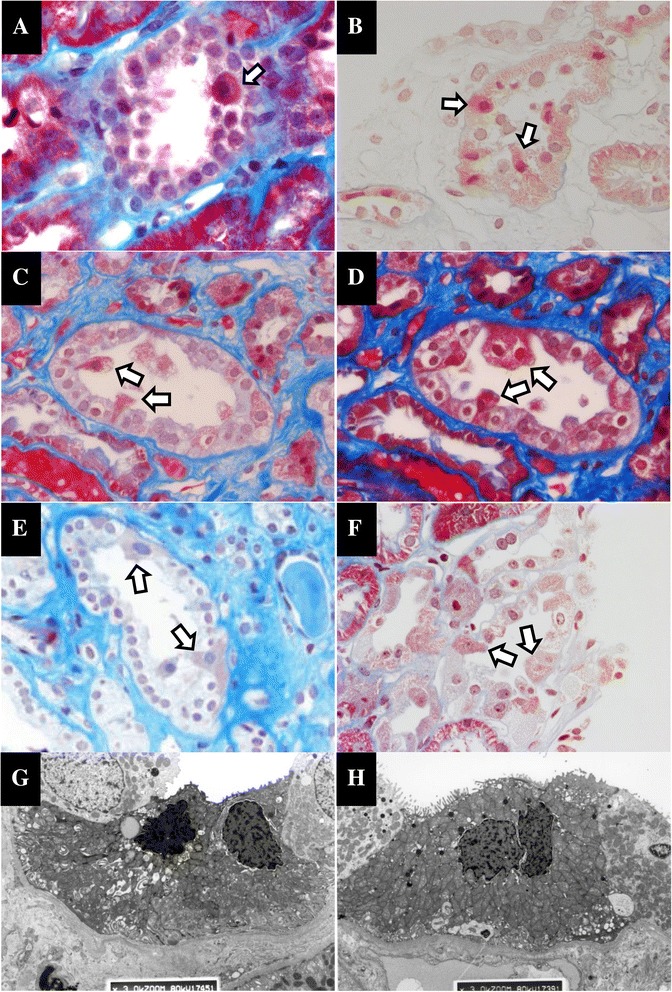
**Photos of tubules in patients of LBW-related nephropathy and patients with mtDNA mutation; (A)-(F): Masson’s Trichrome stain, (G) and (H): electron microscopy (original magnification: × 3000).** GESCs are present in collecting ducts or distal tubules of LBW 1 **(A)**, LBW 2 **(B)**, LBW 3 **(C)**, LBW 4 **(D)**, Mt 1 **(E)**, and Mt 2 **(F)**. In LBW 4 **(G)**, the number of mitochondria with morphological abnormalities is increased in the collecting duct. One nucleus appears to be condensed. In Mt 2 **(H)**, increased mitochondria are also observed in the cytoplasm of collecting duct.

The presence of granular swollen epithelial cells (GSECs) in the collecting ducts or distal tubules was recently reported to be a characteristic pathological change in patients with mitochondria cytopathy [14]. All four patients with LBWN as well as the two patients with mtDNA mutations had GSECs in their collecting ducts and/or distal tubules (Figure 3A-F). In addition, a portion of these GSECs had dropped out of the arrangement of the tubules. The electron microscopy analysis revealed that these GSECs contained an increased number of enlarged mitochondria (Figure 3G, H), and the nuclei occasionally seemed to be condensed.

### The expression of Complex IV exhibited a mosaic pattern of staining in the glomeruli and tubules of both the LBW-related nephropathy patients and mtDNA mutation patients

**Figure 4:**
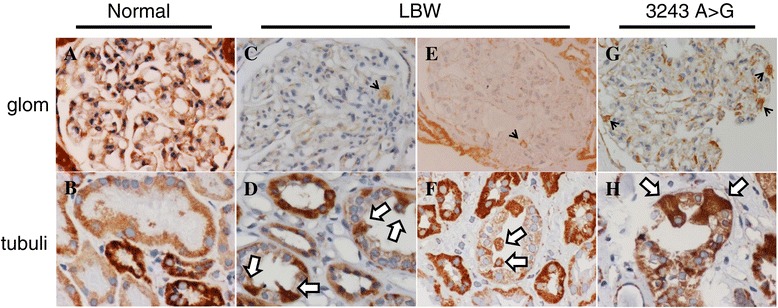
**COX IV staining of the glomeruli and tubules. A**: In the glomeruli of a normal control, the podocytes almost equally express COX IV. **B**. In the tubules of a normal control, the tubular cells at the same luminal level almost equally express COX IV. **C**. In the glomeruli of LBW 1, only one podocyte (arrow) expresses more COX IV than the other podocytes. **D**. In the tubules of LBW 1, unbalanced strong staining for COX IV is detected (arrows). Only a part of tubular cells express extremely intense COX IV compared with other tubular cells at the same luminal level. **E**: In the glomeruli of LBW 4, only one podocyte (arrow) expresses more COX IV than the other podocytes. **F**. In the tubules of LBW 4, staining for COX IV in a part of tubular lumens exhibits a mosaic pattern. The GSECs should express intense COX IV staining (arrows). **G**: In the glomeruli of Mt 1, a portion of podocytes (arrows) express more COX IV than the other podocytes. **H**: In the tubules of Mt 1, a portion of tubular cells are intensely stained (arrows) compared with the other cells in the same lumen. These cells should coincide with GSECs002E.

Complex IV, which is composed of 13 subunits, is the terminal of the electron transfer chain in mitochondria. It was reported that the complex IV activity of podocytes changes in pathological conditions [15]. Therefore, we here stained for COX IV, which is one of the subunits. A COX IV expression analysis of normal controls showed that podocytes should be almost equally positive in the glomerulus (Figure 4A) and that tubular cells are also equally positive at the same luminal level (Figure 4B). However, in the LBWN patients, the expression of COX IV in the podocytes was unbalanced. Only a few podocytes appeared to intensely express COX IV, although the others expressed less COX IV (Figure 4C, E). In addition, the COX IV staining in a part of tubules of the LBWN patients exhibited a mosaic pattern, even at the same luminal level (Figure 4D,F). The cells strongly expressing COX IV appeared to correspond to GSECs. The expression patterns both of the glomerular podocytes and tubules in the patients with mtDNA mutations showed a mosaic pattern (Figure 4G, H), similar to that observed in the LBWN patients. The degree of the mosaic patterns in glomeruli and tubular cells should be more intense in patients with mtDNA mutations compared with LBWN patients.

### The LBW-related nephropathy patients did not have any mitochondrial gene mutations

According to the PCR-Luminex assay, none of the 61 mtDNA mutations were detected in the blood, urine or kidney sections of the four LBWN patients (Table 1). However, an mtDNA mutation (3243 A > G) was clearly detected in all samples of blood, urine sediment and kidney sections in patient Mit 1. Because another patient (Mit 2) had already been found to have 3243 A > G mtDNA at another hospital, we did not perform an mtDNA mutation analysis using RCR-Luminex due to ethical considerations.

## Discussion

Table 2 presents a summary of our results. The characteristic feature of glomerular involvement observed in patients with mitochondrial cytopathy is focal segmental glomerulosclerosis (FSGS), as previously described in a considerable number of reports [9-11]. In addition, it was recently reported that the presence of granular swollen epithelial cells (GSECs), in which enlarged mitochondria increase, in the distal tubules or collecting ducts is a specific pathological change in patients with mitochondrial cytopathy [14]. On the other hand, adults born with LBW have a high risk of kidney damage [1,16] and sometimes exhibit FSGS lesions in their glomeruli [7]. In this report, we showed that the pathological findings of kidney biopsy specimens were similar between patients with mtDNA mutations and those with LBWN with respect to the following three points (Table 2): 1. glomerular changes involve the presence of FSGS lesions and podocytes with foot process effacement; 2. a portion of tubular cells display characteristics of GSECs, in which the number of enlarged mitochondria is increased; and 3. The complex IV expression shows an unbalanced expression pattern in a portion of podocytes and tubular cells. In fact, although our two patients with mtDNA mutations had no FSGS lesions, there is a possibility of sampling error. As another possibility, because these two patients had no other symptoms of mitochondrial cytopathy, the glomerular lesions could be slight. Furthermore, although we evaluated 5 obesity-related nephropathy with FSGS lesions (all patients are under 40-year-old), there were no GSECs.

**Table 2:** **Summary of the findings of nephropathy in the mitochondrial cytopathy and LBW-related nephropathy patients**

	**Normal**	**Mitochondrial cytopathy**	**LBW-related nephropathy**
mtDNA mutation	No	Yes	No
FSGS lesion	No	Yes^1^	Yes
Glomerular hypertrophy	No	Yes or No	Yes
Foot process effacement	No	Yes	Yes
Increased mitochondria in podocyte	No	Yes	No
GSECs in tubules	No	Yes	Yes
COX IV expression in glomeruli	uniform	mosaic pattern	weak (but intense in a few podocytes)
COX IV expression in tubules	uniform	partially mosaic pattern	partially mosaic pattern

^1^Although the two patients with mitochondrial cytopathy had no FSGS lesions in the glomeruli, the presence of FSGS lesions is a characteristic glomerular change in mitochondrial cytopathy patients, as reported in many previous reports.

Because the presence of GSECs in the tubules was previously described to be an exclusive characteristic change in patients with mitochondrial cytopathy (12), we initially suspected that the four patients with LBWN had certain mtDNA mutations. However, based on the PCR-Luminex method [8], which can be used to detect 61 different pathogenic mtDNA mutations, no mtDNA mutations were detected in the blood, urine sediment or kidney sections of the four patients with LBWN. Although these 61 mtDNA mutations include most mtDNA mutations previously reported in Japan [8], we cannot completely deny the existence of other mtDNA mutations in the four LBWN patients. The sensitivity of this method for surveying mtDNA mutations has previously been documented [8]. In addition, this method of detecting mtDNA mutations using blood, urine sediment and kidney sections was able to clearly detect the 3243 A > G mtDNA mutation in patient Mt 1. This report also provides a new strategy for detecting mtDNA mutations in nephropathy patients carrying mtDNA mutations using urine samples. This method is based on the observation that urine sediment consists of certain components derived from podocytes and/or tubular cells.

Why do patients with LBWN exhibit similar pathological changes to those observed in nephropathy patients with mtDNA mutations? It appears that the mitochondrial function of podocytes is involved in the formation of FSGS lesions, as we and others also recently proposed [17-21]. Although we cannot exactly evaluate the degree of foot process effacement by the limitation of sampling, mild foot process effacement were observed all our cases of LBWN. In the present study, we did not observe any increments in the number of mitochondria in the podocytes of the patients with LBWN, which is indeed a different pathology from that observed in mitochondrial cytopathy patients. However, an increased number of mitochondria is only one phenomenon underlying mitochondrial abnormalities and is easily detected pathologically. In fact, mitochondrial biogenesis is controlled by complex mechanisms [22]; therefore, we cannot deny the possibility of mitochondrial dysfunction in LBWN patients, even when an increased number of mitochondria is not observed. COX IV is one of the major components of complex IV, the last enzyme in the respiratory electron transport chain in mitochondria [23]. When we stained the kidney sections using anti-COX-IV antibodies, the staining intensity of podocytes and tubular cells ay the same luminal levels were uniform. However, in patients with mtDNA mutations, COX IV expression, which was mainly expressed by podocytes in glomeruli, showed mosaic patterns. Furthermore, a part of tubular cells with mtDNA mutations extremely expressed COX IV compared with other tubular cells at the same luminal levels (Figure 4). COX IV expression was not uniform in glomeruli of LBWN patients, too. A few podocytes expressed stronger COX IV compared with most of other podocytes. Only a part of tubular cells in LBWN patients expressed extremely intense COX IV as like those in patients with mtDNA mutations (Figure 4). Although we cannot judge whether an unbalanced COX IV expression directly affects the pathogenesis of kidney disease, our results suggest the possibility that the mitochondrial function, not mitochondrial DNA mutations, is associated with the etiopathogenesis of low birth weight-related nephropathy.

In addition, GSECs could be occasionally observed in aged patients. Therefore, GSECs might not be specific findings. However, because all of LBWN patients had GSECs in a part of their tubular cells, we think that GSECs in LBWN should be “meaningful” pathological changes. Furthermore, if GSECs are observed, especially in young patients, the analysis of mtDNA might be needed [24]. Now, we cannot explain why GSECs partially appear. Furthermore, it is obscure that a part of tubular cells show intense COX IV expression although most of tubular cells uniformly express COX IV at the same luminal levels. Although we now hypothesize that unbalance between high energy demand and low energy supply might result in mitochondrial dysfunction, further studies must be needed.

We cannot exclude the possibility that our four patients with LBWN had similar pathological lesions to those observed in the patients with mitochondrial cytopathy “by chance”. In spite of these limitations, some specific cases with characteristic pathological findings should provide new aspects [25-27]. This report provides new clues regarding mitochondria to prompt investigations of the pathomechanisms underlying the renal dysfunction associated with LBW.

## Conclusion

This is the first report to show the pathological similarities not only in glomeruli but also tubuli between nephropathy with a LBW history and nephropathy with mitochondrial cytopathy.

## Abbreviations

LBW: Low-birth-weight
ESRD: End-stage renal disease
CKD: Chronic kidney disease
FSGS: Focal segmental glomerulosclerosis
LBWN: LBW-related nephropathy
COX IV: Cytochrome c oxidase subunit IV
GSECs: Granular swollen epithelial cells
MELAS: Mitochondrial myopathy, encephalopathy, lactic acidosis, stroke-like episodes
